# Canadian colorectal cancer screening programs: How do they measure up using the International Agency for Research on Cancer criteria for organized screening?

**DOI:** 10.1093/jcag/gwae015

**Published:** 2024-05-01

**Authors:** Cindy C Y Law, Li Zhang, André L Carvalho, Linda Rabeneck, Alan N Barkun, Anja Nied-Kutterer, David Armstrong, Clarence K Wong, Diane Lamothe, Donald MacIntosh, Catherine Dubé, Eileen Kilfoil, Jennifer Telford, Nancy N Baxter, Eshwar Kumar, Harminder Singh, Jerry McGrath, Laura Coulter, Daniel Sadowski, Karen Efthimiou, Hendrik DuPlessis, Kelly Bunzeluk, Laura Gentile, Marie-Hélène Guertin, Bronwen R McCurdy, Michael Kohle, Michael Stewart, Ross Stimpson, Scott Antle, Shelley Polos, Steven J Heitman, Tong Zhu, Simbi Ebenuwah, Judy Kosloski, Melissa Mok, Partha Basu, Jill Tinmouth

**Affiliations:** Division of Gastroenterology & Hepatology, University of Toronto, Toronto M5G 1V7, Canada; Division of Gastroenterology, Massachusetts General Hospital, Boston 02114, United States; International Agency for Research on Cancer (World Health Organization), Lyon 69007, France; International Agency for Research on Cancer (World Health Organization), Lyon 69007, France; Dalla Lana School of Public Health and Department of Medicine, University of Toronto, Toronto M5T 3M7, Canada; Division of Gastroenterology & Hepatology, McGill University, Montreal H3A 0G4, Canada; Health PEI, Charlottetown C1A 7N8, Canada; Division of Gastroenterology & Hepatology, McMaster University, Hamilton L8S 4L8, Canada; Division of Gastroenterology, University of Alberta, Edmonton T6G 2R3, Canada; Yukon Government, Whitehorse Y1A 2C6, Canada; Division of Gastroenterology, Dalhousie University, Halifax B3H 4R2, Canada; Division of Gastroenterology, University of Ottawa, Ottawa K1H 8M5, Canada; Nova Scotia Health Authority, Halifax B3S 0H6, Canada; Division of Gastroenterology, University of British Columbia, Vancouver V5Z 1M9, Canada; School of Population and Global Health, University of Melbourne, Melbourne 3053, Australia; Government of New Brunswick, Fredericton E3B 5H1, Canada; Division of Gastroenterology, University of Manitoba, Winnipeg R3A 1R9, Canada; Division of Gastroenterology, Memorial University, St John’s, A1B 3V6, Canada; CancerCare Manitoba, Winnipeg R3E 0V9, Canada; Division of Gastroenterology, University of Alberta, Edmonton T6G 2R3, Canada; Saskatchewan Health Authority, Regina S7K 0M7, Canada; University of Saskatchewan, Saskatoon S7N 5A2, Canada; CancerCare Manitoba, Winnipeg R3E 0V9, Canada; BC Cancer Agency, Vancouver V5Z 4E6, Canada; Institut National de Santé Publique du Québec, Québec G1V 5B3, Canada; CancerCare Ontario, Toronto M5G 2L3, Canada; Government of Nunavut, Iqaluit X0A 0R0, Canada; Division of Gastroenterology, Dalhousie University, Halifax B3H 4R2, Canada; CancerCare Manitoba, Winnipeg R3E 0V9, Canada; Eastern Health Cancer Care, St. John’s, A1B 3V6, Canada; Saskatchewan Cancer Agency, Regina S4W 0G3, Canada; Division of Gastroenterology & Hepatology, University of Calgary, Calgary T2N 4Z6, Canada; Saskatchewan Cancer Agency, Regina S4W 0G3, Canada; CancerCare Ontario, Toronto M5G 2L3, Canada; Saskatchewan Cancer Agency, Regina S4W 0G3, Canada; Faculty of Medicine, University of Calgary, Calgary T2N 4N1, Canada; International Agency for Research on Cancer (World Health Organization), Lyon 69007, France; Division of Gastroenterology & Hepatology, University of Toronto, Toronto M5G 1V7, Canada

**Keywords:** Colorectal cancer, screening, organized

## Abstract

**Background:**

Canada has one of the highest incidences of colorectal cancer (CRC) worldwide. CRC screening improves CRC outcomes and is cost-effective. This study compares Canadian CRC screening programs using essential elements of an organized screening program outlined by the International Agency for Research on Cancer (IARC).

**Methods:**

We collaborated with the Cancer Screening in 5 continents (CanScreen5) program, an initiative of IARC. Standardized data collection forms were sent to representatives of provincial and territorial CRC screening programs. Twenty-five questions were selected to reflect IARC’s essential elements of an organized screening program. We performed a qualitative analysis of Canada’s CRC screening programs and compared programs within Canada and internationally.

**Results:**

CRC screening programs exist in 10 provinces and 2 territories. None of the programs in Canada met all the essential criteria of an organized screening program outlined by IARC. Three programs do not send invitations to participate in screening. Among those that do, 4 programs do not include a stool test kit in the invitations. While all provinces met the essential elements for leadership, governance, finance, and access to essential services, there was more heterogeneity in the domains of service delivery as well as information systems and quality assurance.

**Conclusions:**

There is considerable heterogeneity in the design of CRC screening programs in Canada and worldwide. Programs should strive to meet all the essential IARC criteria for organized screening if local resources allow, such as issuing invitations and implementing systems to track and compare outcomes to maximize screening program quality, effectiveness, and impact.

## Introduction

Colorectal cancer (CRC) is the fourth most common malignancy and the second leading cause of death due to cancer.^1^ In Canada in 2022, there were an estimated 24 300 new cases diagnosed and 9400 deaths attributed to CRC.^1^ The projected age-adjusted incidence rate of CRC for 2022 in Canada is 52.9 per 100 000,^1^ which ranks amongst the highest in the world. Reducing this high burden of disease is important for the health and quality of life of Canadians.

CRC screening decreases CRC incidence and mortality through the detection and removal of premalignant lesions and the identification of earlier-stage cancers.^2–4^ CRC screening can be performed using a variety of non-invasive and invasive methods. Faecal tests such as the faecal immunochemical test (FIT) and the guaiac faecal occult blood test (gFOBT) are safe, non-invasive screening modalities that can be performed at home.^5^ When faecal tests are abnormal, colonoscopy is recommended to visualize the colon to diagnose and remove premalignant lesions and/or CRCs. Flexible sigmoidoscopy^6^ or a colonoscopy alone,^7^ without prior faecal testing, can alternatively be used for primary CRC screening, but these strategies are less commonly performed in Canada. Regardless of the test adopted, it is important to recognize that screening for CRC is a multi-step process. The effectiveness of a screening program depends on the adherence and performance quality of its component steps.^8^

Screening can be broadly categorized into 2 approaches: opportunistic and population-based.^9^ Opportunistic screening occurs at the patient’s request or when recommended by a healthcare provider such as by the patient’s primary healthcare provider during a routine appointment.^9^ Population-based screening programs are centrally designed and managed according to a national or regional screening policy. A defining feature of population-based screening programs is the ability to identify eligible individuals and send them personal invitations to attend the screening.^10^ While being population-based is a key criterion for being an “organized” screening program, programs should also meet several other criteria to truly be considered organized. The International Agency for Research on Cancer (IARC) recently conducted a Delphi process with international experts to define essential and desirable elements of organized cancer screening programs.^11^ They identified 16 essential criteria in 5 categories: (1) leadership, governance, finance (ie, existence of a policy, team, and resources to implement and coordinate); (2) health workforce (ie, training of service providers); (3) access to essential services (ie, adequate infrastructure, workforce, and supplies, as well as equity of access); (4) service delivery provision (ie, system to identify, invite, inform, and recall individuals); and (5) information system and quality assurance (ie, system to identify cancer occurrence, evaluation of performance, and quality).^11^

An organized approach to screening is associated with increased participation and decreased CRC mortality.^12–14^ Screening is more accessible and equitable when administered through an organized program.^9^ Organized programs often also include other benefits such as quality assurance protocols and systems to ensure follow-up of abnormal results.^9^ There is inherently more variation in the process when screening occurs opportunistically; further, there is a greater risk of possible harms (eg, over-screening low-risk individuals or underscreening high-risk individuals).^9^ The value of organized screening was highlighted by the Canadian Task Force on Preventive Health Care in 2016 their statement on CRC screening in asymptomatic adults who are not at high risk for colon cancer.^4^

However, there is significant heterogeneity in the way CRC screening programs are implemented across jurisdictions. Recognizing this issue, Cancer Screening in 5 Continents (CanScreen5) was initiated by IARC to collect information on the characteristics and performance of cancer screening programs worldwide in a harmonized manner with the core objective of encouraging and supporting countries to collect and use cancer screening data for effective program evaluation and quality improvement.^15^ IARC is the cancer agency of the World Health Organization and its mandate is to promote international collaboration in cancer research.^16^ CanScreen5 is an international network initiated by IARC in 2019 to uniformly collect, analyze, and disseminate information on the characteristics and performance of cancer screening programs worldwide. CanScreen5 builds upon IARCs previous successful experience in reporting the status of implementation and performance of cancer screening in EU Member States.^10^

We collaborated with the CanScreen5 team to collect qualitative information on Canadian CRC screening programs. According to CanScreen5, the minimum criteria to be considered a screening program are the existence of a screening protocol and a formal commitment from the government to provide screening services. This study compares Canadian CRC screening programs using data collected for the CanScreen5 initiative including essential elements outlined by IARC to inform existing program management, guide policy-making, and stimulate research.

## Methods

Standardized CanScreen5 qualitative data collection forms were sent to representatives of CRC screening programs in Canada’s 10 provinces and 3 territories in 2020 and 2021. The form consists of 52 questions pertaining to organization of screening, information system and data collection, screening protocol, invitations for screening and further assessment, and quality assurance of screening activities. After the data were received by IARC, they underwent a rigorous 2-step validation process: review by the IARC Secretariat followed by validation by the CanScreen5 Scientific Committee.^10^ Representatives from individual screening programs were contacted for clarification whenever necessary. Finally, the data were uploaded to a publicly available CanScreen5 web portal.^10^

The general characteristics (eg, type of screening performed, target age for screening, and primary screening test used) of each provincial and territorial program in Canada were summarized. We selected 25 questions from the CanScreen5 questionnaire to facilitate comparisons across programs (Table 1). The questions were selected to reflect the key components of an organized screening program as proposed by Zhang et al.^11^ As this framework was published after the creation of the CanScreen5 questionnaires, there were no applicable questions in the survey pertaining to the health workforce (training of service providers) component. Heat maps were created to summarize and depict the findings.

**Table 1.** The Canscreen5 qualitative data questions used, organized by key components of a screening program^12^

Leadership, governance, finance
1. Is there an individual/team/institution responsible for the management/coordination of the cancer screening activities?
2. Does the Health Ministry/Authority allocate a budget to cancer screening?
3. Is there a policy document from the Health Ministry/Authority that recommends cancer screening?
4. Is there a screening protocol or guideline?
5. Is there a defined target age?
6. Is there a defined primary screening test?
7. Is there a defined screening interval?

Access to essential services
8. Are the screening tests available free of charge to the eligible population?
9. Are the diagnostic tests available free of charge to screen-positive individuals?
10. Are treatment services available free of charge to individuals with a diagnosis of precancer/cancer?

Service delivery provisions
11. Are there any initiatives to create population awareness by the Health Ministry/Health Authority?
12. Is there a system to send individual invitations to all the eligible population?
13. If yes to the previous question, does the invitation include a screening kit?
14. Are the screen-positive individuals actively contacted to ensure compliance with further assessment?
15. Are the individuals with a precancer/cancer diagnosis actively contacted to ensure compliance with further management?

Information system and quality assurance
16. Is there a documented guideline/policy for quality assurance of the screening service delivery?
17. Is there an individual/team/institution responsible for quality assurance of the screening service delivery?
18. Is there a system of accreditation of endoscopy units?
19. Is there a system of accreditation for pathology services?
20. Are there specified performance indicators to assess the performance of screening?
21. Is there a computerized information system that collects screening-related data on an individual basis?
22. Is there a system that gathers aggregated data on screening activities?
23. Are screening data linked with population-based cancer registries?
24. Does the programme collect data on the stage of the cancers detected through the programme?
25. Does the programme collect data on the treatment of the precancers/cancers detected through the programme?

To benchmark Canada’s findings with other countries, we selected 3 countries from each continent with the highest incidence of CRC according to GLOBOCAN data^17^ and created heat maps using the same CanScreen5 variables. The countries selected were Australia, Turkey, the Republic of Korea, Japan, Jamaica, Uruguay, Cuba, Hungary, Netherlands, and Denmark. Of note, based on the available data, there is no national colorectal cancer screening programme among participating countries in Africa. In addition, in Oceania, only Australia is currently included in the CanScreen5 network.

## Results

Ten provinces and 2 territories submitted data using the CanScreen5 qualitative data collection form. A representative from Nunavut, which does not have a CRC screening program, was contacted but elected not to submit responses and was excluded from this portion of the study.

The general characteristics of CRC screening programs are summarized in Table 2. The first CRC screening programs in Canada were launched in Alberta, Manitoba, and Ontario in 2007, with other provinces following over time. The newest program, in the Northwest territories, was launched in 2020. As this program is relatively new, population-based screening is currently available in 6 of 7 health regions in the Northwest territories with plans to expand the program to all 7 regions by the end of 2023. The target population for all programs currently are individuals between the ages of 50 and 74. With the exception of Manitoba, the primary screening test used in all Canadian provincial programs is the FIT. Manitoba uses the highly sensitive gFOBT.

**Table 2.** General characteristics of CRC screening programs in Canada

Province or territory	Meets CanScreen5 definition of screening program?^e^	Meets criteria for population-based program?^d^	Launch of screening program	Age range for screening (years)	Primary screening test used	Screening interval (months)
Alberta	Yes	Yes	2007	50–74	FIT^b^	24
British Columbia	Yes	No	2013	50–74	FIT	24
Manitoba	Yes	Yes	2007	50–74	gFOBT^c^	24
New Brunswick	Yes	Yes	2014	50–74	FIT	24
Newfoundland and Labrador	Yes	No	2012	50–74	FIT	24
Northwest territories	Yes	Yes^a^	2020	50–74	FIT	12 to 24
Nova Scotia	Yes	Yes	2009	50–74	FIT	24
Nunavut	No	No	N/A	50–74	FIT	24
Ontario	Yes	Yes	2007	50–74	FIT	24
Prince Edward Island	Yes	Yes	2011	50–74	FIT	24
Quebec	Yes	No	2011	50–74	FIT	24
Saskatchewan	Yes	Yes	2009	50–74	FIT	24
Yukon	Yes	Yes	2017	50–74	FIT	24

^a^Population-based screening implemented in 6 of 7 health regions with the expansion of the program in progress.

^b^FIT: Faecal immunochemical test.

^c^gFOBT: Guaiac faecal occult blood test.

^d^Population-based programs are defined as centrally designed and managed programs with the ability to identify eligible individuals and send them personal invitations to attend screening.

^e^CanScreen5 definition of screening program: Existence of a screening protocol and a formal commitment from the government to provide screening services.

**Figure 1.**
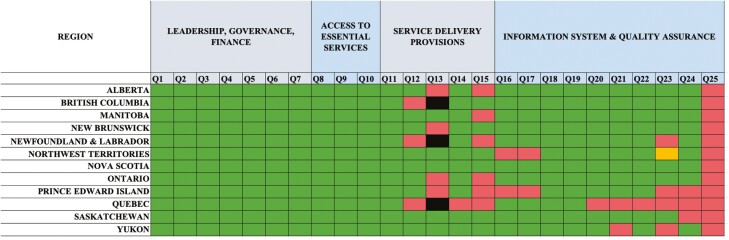
Colour charts depicting selected characteristics of colorectal cancer screening programs/initiatives in Canadian provinces. Green = Yes, Red = No, Orange = Unknown, Black = Not applicable.

Figure 1 summarizes the selected characteristics of Canadian CRC screening programs using a heat map. All the screening programs met leadership, governance, and finance criteria, as well as access to essential service criteria. However, responses were more heterogeneous with regard to service delivery provisions and information system and quality assurance.

None of the provincial programs meet all the essential and desirable criteria to be considered organized. With the exception of Nunavut, all provinces and territories have a screening program that meets the CanScreen5 definition of a screening program. British Columbia, Newfoundland, and Quebec do not meet the criteria of a population-based screening program as they do not send invitations to eligible citizens to participate in screening. Compared to Quebec, British Columbia, and Newfoundland have more sophisticated processes to follow up on results and monitor quality. Among the regions that do send invitations, New Brunswick, Ontario, Prince Edward Island, and Alberta do not include a stool test kit in the invitations. Instead, residents receive a letter inviting them to speak to their primary healthcare provider about CRC screening. With regard to quality assurance, all provinces report that endoscopy units and pathology services are accredited, and nearly all use specific indicators to assess screening performance quality.

The greatest heterogeneity across programs was in the area of information systems and quality assurance. All of Canada’s CRC screening programs collect some data on screening-related outcomes. However, the level of detail of the information gathered (ie, stage of cancers detected and treatment of detected cancers) varies widely. Notably, tracking is more limited in Quebec due to the opportunistic nature of screening in the province.

**Figure 2.**
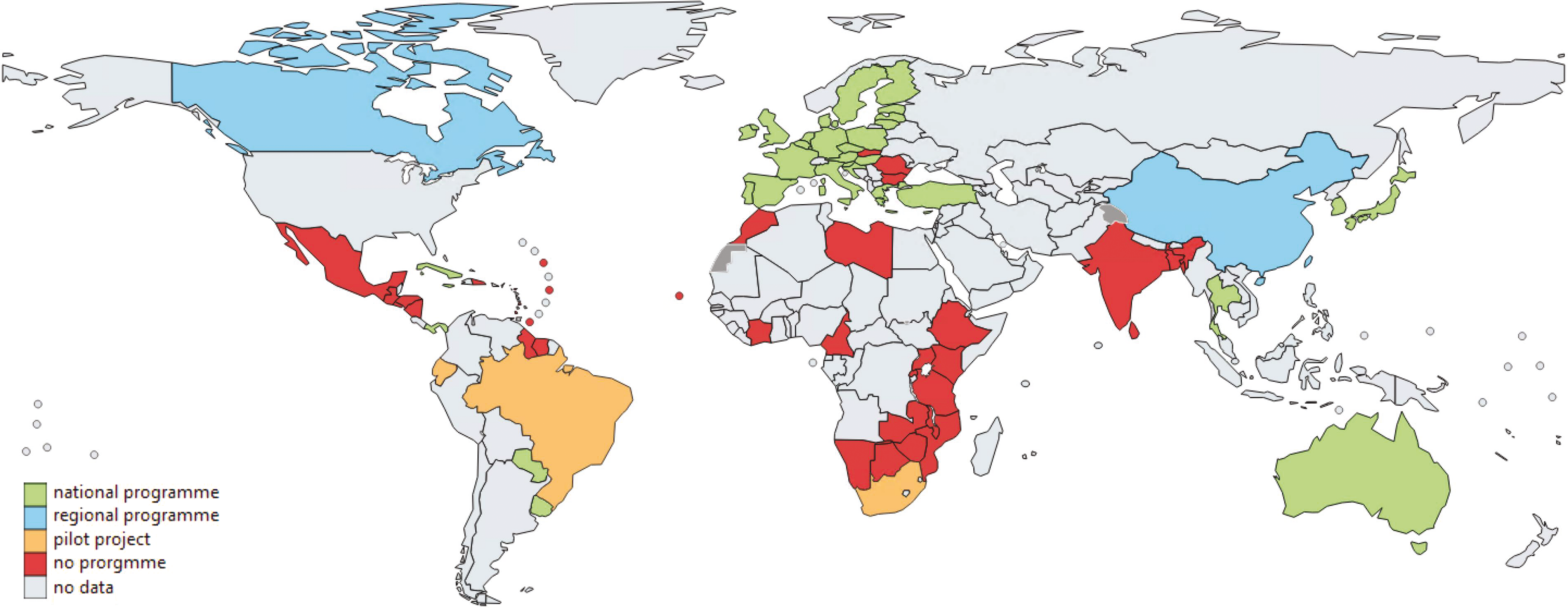
Presence of CRC screening programs around the world according to CanScreen5 data.

**Figure 3.**
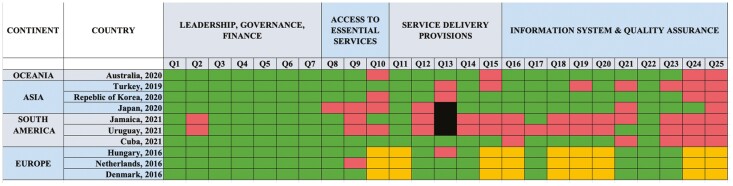
Colour charts depicting selected characteristics of selected colorectal cancer screening programs/initiatives from jurisdictions outside of North America. 3 countries in each continent with the highest incidence of CRC were included. For some continents, fewer than 3 countries were reported due to data availability via CanScreen5. Green = Yes, Red = No, Orange = Missing Dataa, Black = Not applicable. Grey = No program.

Similar to our findings within Canada, there is significant variation across CRC screening programs worldwide. To date, 79 countries have submitted CRC screening information to CanScreen5. Just 37 countries have established CRC screening programs (Figure 2). Figure 3 compares CRC screening programs from the 10 countries reporting to Canscreen5 with the highest CRC incidence by continent. Even among countries with established CRC screening programs, there are important differences in the approach to invitation, following up of results, information systems and quality assurance.

## Discussion

In this study, we performed a qualitative analysis of Canada’s CRC screening programs using data collected via IARC’s CanScreen5 questionnaire. This was done to compare CRC screening programs within Canada and internationally using IARC’s framework of key elements of an organized cancer screening program. We found that none of the Canadian programs fully met all the criteria of an organized screening program. While all Canadian CRC screening programs met the standards for leadership, governance, finance and access to essential services, there was heterogeneity in the domains of service delivery as well as information systems and quality assurance.

Canadian CRC screening programs comprise a mix of longstanding and more recently launched programs. In general, programs evolve and are strengthened over time. More established screening programs such as those in Ontario, Alberta, and Manitoba may be a valuable resource for provinces and territories with newer and smaller-scale screening systems. Opportunities for knowledge exchange include meetings and events organized by groups such as the Canadian Screening for CRC Research Network, and the Canadian Partnership Against Cancer National Colon Cancer Screening Network.

Heterogeneity across programs was observed in service delivery as well as information systems and quality assurance. In particular, only 5 out of 12 jurisdictions send out invitations and kits to participants, despite the fact that studies have consistently shown that invitations are associated with increased participation in screening,^18^ and that the inclusion of kits with the invitations leads to even greater participation.^19^ Further initiatives are required to explore and address the barriers to implementing invitations in provinces where programs have been established (British Columbia, Quebec, and Newfoundland), and to include FIT kits with the invitations in provinces sending invitations but without kits (Alberta, New Brunswick, Ontario, and Prince Edward Island). Furthermore, the role of technology in the delivery of invitations is also an area of potential study. Currently, invitations are mailed to eligible individuals in 7 provinces and 2 territories. However, using multiple communication channels (ie, email, text messaging, or social media advertisements)^20^ may lead to greater awareness and participation in screening, and may facilitate targeted messaging to under or never screened populations.

Currently, the majority of provinces do not link screening data with population-based cancer registries and do not collect data on the treatment of colorectal lesions detected through the program. Without doing so, it is difficult to accurately evaluate the impact of screening on cancer burden, to identify potential areas of program inefficiencies, and to compare outcomes across provinces for quality assurance. Canada’s large geographic size, where delivery of health services is organized by province or territory (each with its own health information systems), also contributes to the challenges with interprovincial comparisons. Initiatives such as Health Data Research Network Canada^21^ have recently launched and could potentially be used to collect harmonized data across the provinces and territories. Investment in information technology and data collection systems to support more robust tracking of outcomes is a potential area for future improvement.

CanScreen5 collects data globally, allowing for standardization of data and ease of comparison across jurisdictions. CRC screening is not available in all countries. Among countries with established CRC screening programs, there are important differences in the approach to invitation, follow-up of results, information systems and quality assurance. While there are established criteria for an ideal screening program, in practice, programs vary around the world. Reasons for this may include the age of the program, the region or country’s health system and the availability of resources to support screening.

The CanScreen5 Interface is convenient to use but does have some drawbacks. Although we were able to readily identify major differences between programs, more subtle differences such as the role of the primary healthcare provider in screening are not captured. Another important consideration is that data from some countries may not be up to date. For example, the CanScreen5 data for European countries is several years old and important program changes may have occurred. However, CanScreen5 is a growing initiative and it is hoped that data collection processes become more robust as partnerships with local CRC screening programs become more established. A final limitation of this study is CanScreen5 questionnaire does not cover the criterion of training of service providers, which was identified as a criterion for an organized cancer screening program based on the Delphi process.^11^ This is an important parameter to measure as there is considerable variation in training across jurisdictions and this variation has implications for the quality of programs. Future iterations of the CanScreen5 data collection tool could consider the addition of questions to address this aspect of the framework.

In conclusion, Canada has made significant strides in establishing CRC screening activities in the past 2 decades. However, none of the programs in Canada meet all the essential criteria of an organized screening program outlined by IARC, and this study highlights areas where action should be considered. First, CRC screening programs should be established in all provinces and territories. Secondly, in provinces and territories where programs are established, invitations should be sent out to all those in the screen eligible, the target population, and FIT should be included with these invitations. Finally, all provinces would likely benefit from implementing systems to track and compare outcomes to maximize their quality, effectiveness, and impact.

## Supplementary Material

gwae015_suppl_Supplementary_Materials

## Data Availability

The data underlying this article are available in the Cancer Screening in 5 Continents (CanScreen5) online database, at https://canscreen5.iarc.fr/?page=factsheets.
